# Risks from accidental exposures to engineered nanoparticles and neurological health effects: A critical review

**DOI:** 10.1186/1743-8977-7-42

**Published:** 2010-12-21

**Authors:** Myrtill Simkó, Mats-Olof Mattsson

**Affiliations:** 1 Austrian Academy of Sciences, Institute of Technology Assessment, Vienna, Austria; 2 Health and Environment Department, Environmental Resources and Technologies, Austrian Institute of Technology, Seibersdorf Austria

## Abstract

There are certain concerns regarding the safety for the environment and human health from the use of engineered nanoparticles (ENPs) which leads to unintended exposures, as opposed to the use of ENPs for medical purposes. This review focuses on the unintended human exposure of ENPs. In particular, possible effects in the brain are discussed and an attempt to assess risks is performed.

Animal experiments have shown that investigated ENPs (metallic nanoparticles, quantum dots, carbon nanotubes) can translocate to the brain from different entry points (skin, blood, respiratory pathways). After inhalation or instillation into parts of the respiratory tract a very small fraction of the inhaled or instilled ENPs reaches the blood and subsequently secondary organs, including the CNS, at a low translocation rate. Experimental in vivo and in vitro studies have shown that several types of ENPs can have various biological effects in the nervous system. Some of these effects could also imply that ENPs can cause hazards, both acutely and in the long term. The relevance of these data for risk assessment is far from clear. There are at present very few data on exposure of the general public to either acute high dose exposure or on chronic exposure to low levels of air-borne ENPs. It is furthermore unlikely that acute high dose exposures would occur. The risk from such exposures for damaging CNS effects is thus probably very low, irrespective of any biological hazard associated with ENPs.

The situation is more complicated regarding chronic exposures, at low doses. The long term accumulation of ENPs can not be excluded. However, we do not have exposure data for the general public regarding ENPs. Although translocation to the brain via respiratory organs and the circulation appears to be very low, there remains a possibility that chronic exposures, and/or biopersistent ENPs, can influence processes within the brain that are triggering or aggravating pathological processes.

In general, the present state of knowledge is unsatisfactory for a proper risk assessment in this area. Crucial deficits include lack of exposure data, the absence of a proper dose concept, and that studies often fail in adequate description of the investigated ENPs.

## Introduction

The purpose of the present review is to give a short overview of how engineered nanoparticles (ENPs) can translocate from the respiratory tract to the circulation, pass the blood-brain-barrier (BBB), affect the brain, and to discuss possible adverse health effects and associated risks. We also suggest that there is a need for focused research to support risk assessment. This research should use standardized and proper methods and experimental designs including the selection of the right in vitro and/or in vivo models, controls, ENP characteristics, doses, etc.

Nanoparticles (NPs) can be generated through both natural (e.g., combustion by-products, volcano eruption etc.) and synthetic processes. In the present article, we focus on engineered nanoparticles and their unintended exposure of the CNS.

In principal, researchers have agreed to use the term nanoparticle if the material size is smaller than 100 nm in three dimensions and are singular particles; although different terms are still used in the literature, like nanosized materials, ultrafine particles (UFP), engineered nanomaterials, manmade nanoparticles [1]. This shows that the expression "nanomaterial" is related to the size dimension only, but not to the material itself which can contain any kind of substance. This is relevant from different perspectives, e.g. in political discussions and decisions but also for dosimetry aspects. For the latter, it is important to characterize the kind of the nanomaterial, to define concentration(s), establish dose response relationships etc. Dosimetry is furthermore necessary for risk estimation and for the establishment of thresholds and/or limit values. The general use of the term nanomaterial does not say much about the chemical conditions. Therefore, the physico-chemical properties have to be known for exposure calculations, including size, shape and composition of the material.

## ENPs and dose

For the calculation of the biological or chemical reactivity of the material, knowledge about the physico-chemical properties, the number of molecules on the surface of the nanosized material is needed, as well as the number of particles per cell. The number per cell is important to determine the **effective dose**, since nanoparticles have larger surface area than the corresponding bulk material including a higher number of molecules on the surface which can interact with the biological material, and their larger number per mass allows their dispersion into more cells. Information about the physico-chemical properties including size and shape are important in order to estimate ENP specific effective dose as well. In in vitro studies it is difficult to estimate this dose because NPs diffuse, settle, and agglomerate in cell culture media depending on different factors like media density and viscosity, particle size, shape, charge and density. Teeguarden et al. [2] developed a particokinetic model to estimate cellular dose in vitro considering different factors like the dynamic precipitation rate in cell culture media which depends on particle size and follows more the Brownian motion than gravitation.

Another important dose measure might be the **relative biological effectiveness (RBE)**. If the physico-chemical properties and the number of molecules on the surface of the nanosized material are known, as well as the number of particles per cell, weighting factors might be introduced as in dose calculation for ionizing radiation, where RBE is calculated as a function of the quality of the radiation. Thus, for the same absorbed dose, alpha radiation is 20 times more biologically potent than x-rays or gamma radiation. Accordingly, RBEs can then be calculated for specific nanomaterials. The RBEs would then be dependent of the material itself and the number of internalized/taken up nanoparticles per cell. The so called biological effective dose (BED) concept describes oxygen radical generation, as an indirect measure (or marker) for BED [3] considering the physico-chemical properties of the material. This concept is very useful. However, specific cell type dependent redox potential capacities have also to be considered. This is reviewed by Valco et al. [4] where pH dependent effects are shown to be due to the specific redox capacity of the cell type in question, with cell type dependent effects on e.g. cell cycle and developmental events. In addition, the work by Sohaebuddin et al. [5] shows such cell type dependent effects of various ENPs. Furthermore, for dose calculations relevant for both chronic and for acute exposure, the **dose rate **has to be known, which includes the time factor. To determine the retention time (how long an ENP is present in a cell or a body) of a certain ENP, knowledge about the physico-chemical properties, but also about the biological deposition time in each site (deposition and retention time are depending on deposition site) is needed. In other words, knowledge about site dependent retention time and bioavailability is needed to calculate the time factor for dose rate. Other factors that will affect the dose rate are that certain ENPs are biodegradable with a relatively short half-life whereas others will not be metabolized within the body. In addition, some ENPs may be excreted, whereas others may accumulate over time. The present knowledge regarding the different dose concepts relevant for nanomaterials is however very limited, with possible exception of data from a few in vitro studies. Relevant data from in vivo situations is mainly absent.

## Drug delivery systems and the blood-brain-barrier

ENPs have the potential to revolutionize medicine because of their ability to reach and to affect target organs and tissues, even "as distant" as tumours in the brain, at the molecular and cellular levels. Medical and pharmacological research is focused on applications of nanosized materials, whereas side effects associated with their use are generally not taken into consideration. In fact, the knowledge about potential toxicity of ENPs is far from comprehensive [6,7].

Drug delivery systems or nanocarriers should and may overcome solubility or stability issues for the drug, and minimize drug induced side effects. However, the nanomaterials themselves can also induce significant toxic effects (for reviews see [8,9]). Besides the chemical properties, this can be due to their electric, optical, and magnetic properties that are related to physical dimensions, but also the surface of the material can be involved in catalytic and oxidative reactions which themselves can induce cytotoxicity. This toxicity can be greater than that of a bulk material because the surface area-to-volume ratio for nanomaterials is much greater. Moreover, some nanomaterials contain metals or compounds with known toxicity, and thus the breakdown of these materials could elicit similar toxic responses.

A number of questions pertaining to the safety of nanomaterials in this context are thus obvious. What is the ultimate fate of the drug delivery systems/nanocarriers, and their components within the body? What happens with those which are not bio-degradable and those which are functionalized, like carbon nanotubes, or coated with different agents? Further on, what are the consequences after long term exposure?

The blood-brain barrier (BBB) protects the central nervous system from potentially harmful xenobiotics and endogenous molecules (for review see [9]). The BBB, formed by brain capillary endothelial cells linked together by tight junctions, together with adjacent processes from astrocytes, restricts the transfer of most substances from the bloodstream into the brain. Therefore, substances may gain access to the central nervous system by (lipid-mediated) free diffusion or potentially by receptor-mediated endocytosis. Since tight junctions in the BBB have a gap of only 4-6 nm, it has been suggested that nanoparticles pass through the endothelial cell membrane rather than via inter-endothelial junctions [10].

**Figure 1:**
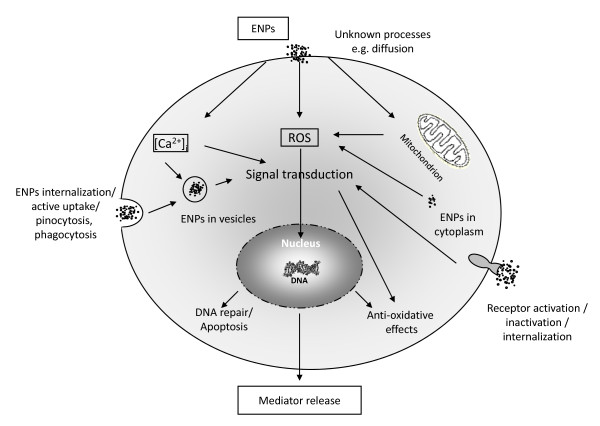
**Various ways for uptake of ENPs to mammalian cells and the effects ENPs can have on intracellular processes**. ROS: reactive oxygen species.

It has been shown that nanoparticles from the blood circulation may influence endothelial cell membrane integrity and/or disrupt the BBB [11], and may induce vesicular transport to gain access into the CNS (see below). Moreover, it seems to be accepted that nanoparticles can induce oxidative stress leading to the generation of free radicals that could disrupt the BBB and cause certain dysfunctions. It is also known that nanoparticles without a surfactant coating are mainly internalized by phagocytes and are thus unable to reach the brain in desirable quantities, therefore almost no pharmaceutical can reach the brain tissues by administering it with uncoated nanoparticles [12]. (See Figure 1 for a description of how ENPs can enter the cell and exert different actions) However, surface modifications of nanoparticles are presently intensely studied for nanomedicinal applications like diagnosis and therapy aiming to influence the target-oriented pharmacokinetic behaviour of nanocarriers. Nanocarriers require surface modifications or other forms of functional modifications for receptor-mediated transport through the brain capillary endothelium to deliver drugs to the central nervous system. Different approaches to obtain suitable modifications are discussed [13]. Kreuter et al. [14] demonstrated that polysorbate 80-coated polybutylcyanoacrylate (P80-PBCA) nanoparticles can deliver the peptide "dalargin" into CNS to induce its analgesic effects. Coating with alternative surfactants did not produce the expected effects [15]. P80-PBCA nanoparticles can thus deliver drugs to the brain, however these nanoparticles seem to have limitations due to their potential toxicity [16]. Thus, Calvo et al. [17] showed a drastic increase in sucrose permeability (as a sign of BBB permeabilization) of the BBB in rats following intravenous administration of P80-PBCA.

Other functional modifications of ENPs include for example the conjugation of cell surface ligands or of antibodies. In order to facilitate the crossing of the BBB, apolipoprotein-E coating of nanoparticles for LDL receptor mediated endocytosis in brain capillaries has also been discussed [15,18]. Apolipoprotein, especially ApoE combined with nanoparticles, behaves like low-density lipoprotein (LDL) in the sense that LDL receptor-mediated transcytosis enhances the drug delivery together with nanoparticles across the BBB and is very effective in drug delivery [19,20].

Another important factor in penetration of the BBB by nanoparticles is their electrostatic charge. Cationic charged molecules occupy anionic areas at the BBB endothelium [21] and increase the endothelial cell permeability [22]. In in vitro studies, the cationized nanoparticles translocate more readily to the brain compared with anionic or neutral nanoparticles [23]. Thus, both the size and the charge of colloidal drug carriers are important factors in determining drug or nanoparticle delivery across the BBB or in brain parenchyma [24]. However, there are little in vivo data regarding brain permeability of cationized nanoparticles. One exception is the work by Lockman et al. [25,26] who investigated the effect of neutral, anionic and cationic charged ENPs on the blood brain barrier (BBB) integrity and permeability in situ with rat brain perfusion. Neutral ENPs and low concentrations of anionic ENPs were found to have no effect on BBB integrity, whereas high concentrations of anionic ENPs and cationic ENPs disrupted the BBB structure. Especially cationic NPs displayed an immediate toxic effect. It has been suggested that the BBB disruption by cationic nanoparticles may be due to opening of inter-endothelial routes, i.e., widening of tight junctions [21]. The structural changes in tight junction may occur without any decrease in the high electrical resistance of the cerebral endothelium. Since cationic nanoparticles are possibly more neurotoxic than other forms, it seems that neutral and low concentrations of anionic nanoparticles are better suited as colloidal drug carriers for enhanced delivery to the brain [26].

Kim et al. [27] synthesized silica-coated magnetic nanoparticles containing rhodamine B isothiocyanate within a silica shell of controllable thickness (50 nm). After intraperitoneal administration into mice (10 mg/kg), ENPs were detected in the mouse brain, indicating a blood-brain barrier penetration without disturbing its function or producing apparent toxicity. In another study they demonstrated that poly(methoxypolyethyleneglycol cyanoacrylate-co-hexadecylcyanoacrylate) (PEG-PHDCA) nanoparticles have the capacity to diffuse through the blood-brain barrier after intravenous administration. The authors suggested that the LDL receptor-mediated pathway was involved in the endocytosis mechanism [28].

It has to be pointed out, that nanoparticles administered intravenously are rapidly cleared from the blood stream by the mononuclear phagocyte system and mainly accumulate in liver and spleen [29]. Specially prepared ENPs with surface modifications using either PLA, PLGA or PEG, or combinations of all, as well as functionalized pegylated PLA/PLGA nanoparticles seem to offer possibilities for drug delivery to the brain. It seems that these special ENPs are more biocompatible and are having a better safety profile, and can furthermore pass the BBB without inducing substantial toxicity even at very high doses (440 mg/kg in mice, [13,30]. This suggests that the possibility for ENP uptake in the CNS is very complex and therefore it is not likely that inhaled or ingested ENPs are reaching the CNS in significant amounts. Furthermore, many NPs are agglomerates or covered by a protein corona, undergoing a fast metabolism and/or excretion. It is known that certain ENPs need surface coating to be internalized by phagocytes which in turn induces oxidative stress by the generation of free radicals. The question arises how long this oxidative stress is present within the CNS and to if and what kind of dysfunctions it is leading? It is very likely that the effects (chronic or acute) are dose dependent, therefore a dose definition is strongly needed for the purpose of risk assessment.

## Translocation of nanoparticles from the respiratory tract to the CNS

Since inhalation is one of the main portals of ENP entry into the body and the majority of knowledge is available on that field we focus on uptake of ENPs in the lungs by inhalation or instillation followed by retention and distribution to secondary organs. Epidemiological and toxicological studies have shown that inhalation and subsequent deposition of ambient ultrafine particles (UFP, < 100 nm) into the lungs have adverse health effects, especially respiratory and cardiovascular effects (e.g. [31,32]). These particles also seem to have effects on CNS properties and functions, as shown by both experimental (e.g. [33-35]) and epidemiological studies [36]. UFPs dominate outdoor particle number concentration and particle surface area and are therefore also capable of carrying large concentrations of adsorbed or condensed toxic air pollutants [37]. The existing information regarding health effects of UFP can possibly be used for predicting some hazardous effects of ENPs. It is known that inhaled particles are size dependently deposited in three different regions, namely the nasopharyngeal, tracheobronchial and in the alveolar region of the respiratory tract. Different studies have shown that 90% of the smaller particles (1 nm) are deposited in the nasopharyngeal and the rest in the tracheobronchial region (for review see [38]). Particles in the range of 1-5 nm deposit in nasopharyngeal, tracheobronchial and in the alveolar region, whereas 20 nm ENPs deposit to around 50% in the alveolar region [39]. Larger particles (0.5-10 μm) are remaining on the epithelial surface in airways and alveoli [40]. The retention time seem to depend on the deposition site. For microparticles (0.5-10 μm) the retention time is 24-48 h in rodent airways [41] and it is likely that this is increasing in humans because of the airway length. Kreyling and colleagues have shown [42] that 75-80% of ENPs (< 100 nm) were long-term retained in the alveolar region where particles are interfering with or within cells, like epithelial cells and macrophages, but also with the serous lining fluid (mucus).

The alveolar region of the lungs is the most permeable since gas exchange between blood and air is taking part here. The air-blood barrier in this region is approximately 2 μm thick [43]. If particles are deposited in a certain area they will be either dissolved and/or metabolized, undergoing clearance mechanisms, or insoluble particles will be enriched in particular areas or even in individual cells of the lungs causing biological or toxicological effects [40,43]. ENPs can pass through the interstitium and can be taken up by epithelial cells. However, Geiser and Kreyling [40] summarized that the main pathway for particle clearance in airways, for any kind of particles, is towards the larynx via mucociliary clearance. The authors pointed out that even particles that were relocated into the underlying interstitium re-appear again on the lung surface to be cleared this way.

Alveolar and airway macrophages are on the inner surface and within the lining layer of the lungs, and are constantly exposed to inhaled particles. Phagocytic uptake is the main mechanism to remove insoluble inhaled microsized particles. Monocytes/macrophages are also circulating within the body to take part in the main pathway for monocytes/macrophage-associated particles clearance, which is the mucociliary transport. It cannot be excluded that circulating particle-containing macrophages may re-enter the interstitium and/or lymph nodes and thus the lymphatic system. On the other hand, it is suggested that inhaled and deposited nanoparticles are not efficiently taken up by surface macrophages. Therefore passive mechanisms like diffusion, adhesive interaction, and also pinocytic uptake are currently discussed for translocation [40,43]. Particles which penetrate cells may enrich, interact with organelles, cause oxidative stress, induce different cellular signalling pathways and leading to cellular effects like the release of inflammatory intermediates such as cytokines and free radicals (see also Figure 1). Mühlfeld et al. [44] have shown that inhaled aerosols of 20 nm TiO_2 _in rats were distributed after one hour to all lung compartments in proportion to the compartment volume, and some particles were detected in erythrocytes within the pulmonary capillaries. Peters et al. [45] hypothesized that the way how particles translocate to secondary organs is by the blood circulation. Nemmar et al. [46] documented by using technetium-labeled carbon NPs by an inhalation study in humans, that a certain amount of NPs diffuse rapidly into the systemic circulation. However, certain published studies report that translocation rates for NPs into the blood circulation are very low [47].

Clearance mechanisms in airways and alveoli are reducing the retention time of NPs in the lungs, therefore only relatively few nanosized particles can translocate to secondary organs. Chen et al. [48] have shown that intratracheal-instilled polystyrene particles with an average diameter of 56.4 or 202 nm, are passing into the blood circulation, but this translocation is between 1-2.5% independently of the particle size. Liu et al. [49] investigated the overall toxicity of nasally instilled nanoscale copper particles (23.5 nm) in comparison with micro-sized copper particles (17 μm) in mice and found only in the high-dose group (40 mg/kg, three times per week) significant pathological changes. It has to be pointed out, that this is an enormously high dose without any physiological significance. These kinds of experiments are useful only for toxicity tests or for hazard identification but not for risk assessment. There are several studies performed using different nanomaterials and sizes and concentrations showing translocation to secondary organs, basically to the liver, spleen and kidney (detailed below). Kreyling et al. [50] performed an iridium (2-4 nm) and/or carbon (5-10 nm) ENP inhalation study with rats to learn about the translocation rate from lungs to blood circulation and secondary organs. The authors detected from 0.1 to 1% Ir-NPs of the retained fraction in liver, spleen, kidneys, heart, and brain, and 1-5% in the remaining carcass (soft tissue and bone). The mixed fraction of Ir with the carbon ENP retained in secondary organs at significantly lower levels than pure Ir-NP. Furthermore, 80 nm aggregates translocated and accumulated significantly less than the 20 nm ones. In a recent review, Geiser and Kreyling [40] summarized the evidence for translocation of certain ENPs like gold, silver, TiO_2_, polystyrene and carbon nanoparticles in the size range of 5 - 100 nm across the air-blood barrier from animal studies. In summary, the translocation fraction out of the lung seems not to exceed 5% for any of the investigated ENPs.

Mills et al. [51] investigated the extent to which inhaled radioactive labelled carbon nanoparticles (Technegas, ^99m^Tc, 4-20 nm, aggregates ca. 100 nm) were able to access the systemic circulation on human volunteers. The authors detected more than 95% of Technegas retention in the lungs, with no accumulation in liver or spleen and concluded that the majority of carbon nanoparticles remain within the lung up to 6 h after inhalation and do not pass directly from the lungs into the systemic circulation. Nemmar et al. [46] showed that 20% of initial lung radioactive carbon nanoparticles were detected in the liver, meaning that 80% remained in the lung.

The translocation rate from the respiratory tract to the central nervous system has been shown to be very low. It is questionable if the amount of nanomaterials which reaches the brain can cause hazardous effects. However, Chen et al. [48] reported, that pulmonary inflammation induced by instillation plays the major role in enhancing the extrapulmonary translocation of particles (using LPS coated particles). This fact is indicating that at least LPS coated nanomaterials can induce inflammatory effects which themselves are changing the microenviroment leading to higher translocation rates to secondary organs [35]. A systemic inflammation can contribute to local inflammation in the brain which in turn can lead to enhancement of ongoing inflammatory reactions in the brain [52].

If ENPs are injected or translocated to the blood circulation, proteins are associating with the nanoparticles, which in turn can lead to an in vivo response [53]. It has been shown that a so called "corona" on the surface of the ENPs is the result of the adsorption of different serum/plasma proteins on ENPs. Cedervall and colleagues [53] reported that proteins compete for the nanoparticle "surface," and the resulting "corona" largely defines the biological identity of the particle. Lundqvist et al. [54] have shown that the nature of the corona is determined by the local chemical property of the nanomaterial including size and surface properties. Therefore the kinetics of the ENPs are also depending on the local corona-structure which is different in each microenviroment. In drug delivery studies using polysorbate 80-coated nanoparticles, it was shown that the ENP adsorbs apolipoproteins from the blood after injection. These particles mimic lipoprotein particles which could be taken up by the brain capillary endothelial cells via LDL receptors [15].

In conclusion, the translocation rate of deposited ENPs from the lung to the blood circulation and then to secondary organs seems not to exceed 5%. Furthermore, the translocation from the blood to the CNS is lower than 1% according to available studies (see [50,55] and Table 1). Corona formation can change the translocation rate and possibly increase the hazardous effects.

**Table 1:** Translocation of various ENPs via respiratory pathways or via injection to blood and/or CNS

Material	Administration			ENP size (nm)**	Translocation to blood	Translocation to CNS	Ref.*
	**Inhalation**	**Nasal instillation**	**Injection**				

Carbon particles	X			4-20	X		[51]

	X			100	X		[46] (human)

		X		36		X	[38]

Cu	X			23.5	X		[49]

Ir	X			2-4	X	X	[50]

MnO_2_	X			30	X	X	[56,59]

	X			23	X	X	[62]

Polystyrene	X			56.4	X	X	[48]

	X			202	X	X	[48]

TiO_2_	X			20	X		[48]

		X		80, 155 (very high doses)		X	[61] (mouse)[64] (mouse)

			X	25-70 (s.c.)		X	[65]

			X	25-70 (i.v.)		X	[66]

			X	5 (i.p.)		X	[67]

Latex particles		X		20-200		X	[57,58] (guinea pigs)

Ag		X		70-110		X	[60]

* Studies were performed on rats unless otherwise indicated.

**s.c. = subcutaneous injection; i.v. = intra venous; i.p. = intraperitoneal

## Axonal transport of ENPs to the brain

An important mechanism of particle endocytosis involves the uptake by sensory nerve endings embedded in airway epithelia. In the nasal region it is the olfactory and trigeminus nerve system, and in the tracheobronchial region it is the extensive sensory nerve network. Translocation to ganglia and the CNS can then be accomplished by axonal transport.

The olfactory nerve pathway may be a critical portal of ENP entry to the central nervous system of humans, especially under high environmental or occupational ENP exposures but also under chronic exposure.

Using colloidal gold particles (50 nm) that were intranasally instilled in monkeys it was shown that particles translocate in the axons of the olfactory nerves to the olfactory bulbs, where nanoparticles were seen in the mitochondria but not in the cytoplasm (as cited by [56]). A study by Hunter and Dey [57] in rats demonstrated the translocation of intranasally instilled rhodamine-labeled microspheres (20-200 nm) to the trigeminal ganglion inside the cranium via uptake into the ophthalmic and maxillary branches of the trigeminus nerve. In another study, Hunter and Undem [58] instilled similar particles intratracheally into guinea pigs and reported a neuronal translocation to the ganglion nodosum in the neck area, which is integrated into the vagal system. More recent studies indicated that neuronal translocation pathways are also operational for other inhaled ENPs. Inhalation of elemental ^13^C ENPs (36 nm, 160 μg/m^3^) resulted in a significant accumulation of these particles in the olfactory bulb of rats on the first day, which constantly increased further throughout day seven after the initial 6 h exposure [38]. Results from another inhalation study with solid nanosized manganese oxide particles (30 nm, 500 μg/m^3^) in rats also demonstrated an increase of particles in the olfactory bulb. Inhalation exposures were for 6 h/day, 5 days/week for up to 12 days. After 12 days of exposure with both nostrils patent, Mn concentrations in the olfactory bulb increased 3.5-fold (from 0.5 to 1.75 ng Mn/mg tissue), whereas lung Mn concentrations doubled; there were also increases in striatum, frontal cortex, and cerebellum. When one nostril was occluded during a 6-h exposure, the accumulation of Mn was seen only in the olfactory bulb of the open nostril [56,59]. These observations suggest that nanoparticles in the air can enter into the CNS via the olfactory nerve during accidental or prolonged environmental or occupational exposure to humans.

Another study showed that inhaled 20 nm nanogold particles (2 × 10^6 ^particles/cm^3^) can accumulate in the olfactory bulb of rats [60]. The exposure for 5 days resulted in a significant increase of gold ENPs in the olfactory bulb (8 ng Au/g body weight). After 15 days of exposure, significant accumulations of gold particles were detected in the septum and entorhinal cortex. Both brain structures receive direct neuronal projections from the olfactory bulb, and are important in attention and new memory formation.

After nasal instillation (500 μg of TiO_2 _nanoparticle suspension every other day for 30 days), the micro-distributions of TiO_2 _NPs (80 nm) and fine TiO_2 _particles (155 nm) in the olfactory bulb of mice were investigated by [61]. It could be demonstrated that both types of investigated TiO_2 _particles were taken up by the olfactory bulb via the primary olfactory neurons and then accumulated in the olfactory nerve layer, olfactory ventricle, and granular cell layer of the olfactory bulb. The TiO_2 _content was increased in all investigated brain regions (olfactory bulb, cerebral cortex, hippocampus and cerebellum), with the most significant increases seen in the hippocampus. The presence of TiO_2 _in hippocampus was furthermore accompanied by changes in neuron morphology and increased amount of GFAP-positive cells in the CA4 region. Signs of oxidative stress were documented in all regions of the brain. Interestingly, in general anatase TiO_2 _gave rise to stronger effects than the rutile form.

**Figure 2:**
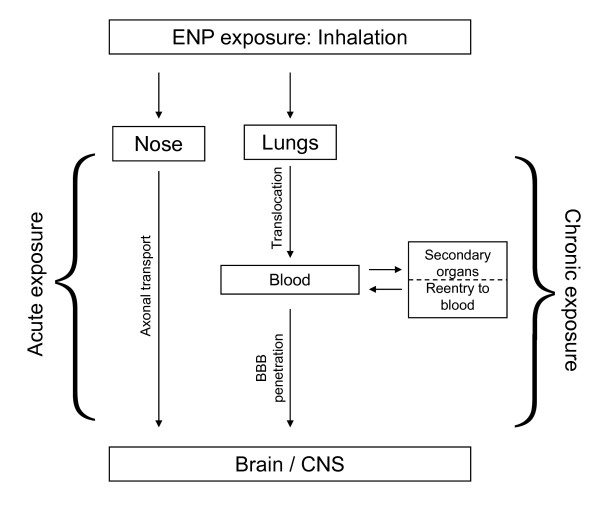
**Overview of the routes by which ENPs can translocate after inhalation through the nose or the lungs to the brain**. Note that inhalation through the nose represents the likelihood of acute exposure effects whereas the inhalation pathway through the lungs followed by translocation to secondary organs and possible re-entry to the blood is showing the probability for chronic exposure.

Taken together, it seems that nanoparticles can translocate to the nervous system through sensory nerves. Translocation of 20 nm particles is 2-10 times higher in the human olfactory bulb than in rats [6]. Thus, the translocated nanoparticles in humans can enter into the deeper brain structures in short exposure time. Based on the limited data available, it is presently difficult to assess to what extent accumulation in the brain via axonal transport is a realistic possibility (see also Figure 2).

## Neurobiological effects of ENPs

### In vivo studies

Of the two principal cell types in the nervous system (neurons and glia cells), the neurons have characteristics that make them especially sensitive to various types of stressors. The neurons have an especially vulnerable anatomy due to their extensive and very thin and fragile extensions (dendrites and especially axons). In addition, these cells are metabolically very sensitive since they rely solely on aerobic metabolism of glucose. The neurons are extremely sensitive to oxidative stress, which in many cases also is a contributing factor to a number of neurodegenerative diseases. In addition, with very few exceptions, neurons are not renewable in mammals, making the nervous system functions very sensitive to agents that cause cell death.

Studies performed on intact animals can specifically address both exposure and dose requirements for potential effects, as well as the specific effects on processes in the brain (Table 2).

**Table 2:** Experimental findings of neurobiological effects of specific ENPs

End-point	In vivo	In vitro	References
Cell morphology changes	Al_2_O_3_, TiO_2_	Fe_2_O_3_, QDs	[11,61,67,69,70]

Increased inflammation signs and markers	MnO_2_		[56,62]

Increased oxidative stress	TiO_2_, QDs	Degussa P25, ferritin, C60, Ag	[61,67,69,72]

Antioxidative effects (inconsistent effects)		CeO, YO, C60	[73-76]

Neuron function (inhibition and facilitation)	MnO_2_, Zn, TiO_2_, QDs	Mn, Ag, ZnO, CuO, CNTs, TiO_2_, QDs	[61,67,68,77,88]

Behaviour (negative effect)	MnO_2_		[62]

Development and differentiation (inconsistent effects)	TiO_2_	Fe_2_O_3_, Ag, TiO2	[65,66,84]

Accelerated protein fibrillation		TiO_2_, CNT, QDs, CeO, copolymer particles	[86,87]

That nanosized MnO_2 _NP can translocate into the brain after long-term inhalation/instillation has been shown by Elder et al. [56] and Sarközi et al. [62]. In the former study, rats were exposed to MnO_2 _ENP by inhalation for 12 days. Mn levels were seen to increase in the olfactory bulb (3.5 times the background levels), striatum, frontal cortex, and cerebellum. No functional endpoints were investigated in this study, but molecular signs of inflammatory changes were possible to detect. In the work by Sarközi and co-workers [62], male Wistar rats were subjected to MnO_2 _(23 nm) instillation for 3, 6, or 9 weeks at 2.63 or 5.26 mg/kg bodyweight. After the end of exposures, Mn was detected in the brain by x-ray spectroscopy. The rats' spontaneous motility was negatively affected. In addition, electrophysiological changes in cortical activity and the conduction velocity of the tail nerve were documented.

Chen et al. [11] infused rats intravenously with Al_2_O_3 _NP (8-12 nm; 29 mg/kg). The rats were sacrificed 20 h after the infusion and the brains were subsequently investigated with immunohistochemistry for certain tight junction proteins normally present in the endothelium of the BBB. The data indicate that the proteins claudin-5 and occludin are down-regulated in the vessels of treated animals, suggesting impairment of the BBB. However, the data are only qualitatively expressed, no proper quantification of the protein levels were made. It is also unclear how many animals were investigated.

In a recent paper by Viswaprakash et al. [63] rat olfactory epithelia were exposed to 1-2 nm zinc particles and the responses to odorants were measured by electroolfactogram and whole-cell patch clamp. The addition of the Zn particles to the odorant suspension enhanced the response to the odorant. Interestingly, this response was seen to be specific for Zn particles, whereas neither Zn^2+ ^ions nor other metal particles (Cu, Ag, Au) elicited similar responses.

The increased production and presence of nanosized TiO_2 _particles in consumer products and in processes has generated an interest into the possible effects of these particles on human health. Regarding in vivo studies of nervous system function, a few recent articles have rendered relevant data. Thus, Wang et al. [61] subjected female mice to nasal instillation with TiO_2 _NP (80 nm, rutile and 155 nm anatase; 500 μg every 2^nd ^day for 30 days) at an extremely high dose. Titanium particles were mainly accumulated in the cerebral cortex, thalamus, olfactory bulb and hippocampus, (especially in the CA1 and CA3 regions). There was an obviously dispersed arrangement of neurons in the hippocampal CA1 region after TiO_2 _exposure. Furthermore, the investigation of cell numbers in the stratum pyramidale of the CA1 region indicated a drastic neuronal loss. There was 30% and 25% cell loss in the 80 and 155 nm TiO_2_-exposed groups, respectively. Apparent morphological changes of hippocampal neurons and increased GFAP-positive astrocytes in the CA4 region were also found, which were in good agreements with the high TiO_2 _contents in this hippocampus region. GFAP is best viewed as a biomarker of early pathological effects, indicated by the activation of astrocytes. Oxidative stress such as lipid peroxidation, protein oxidation and increased activities of catalase, as well as excessive release of glutamic acid and nitric oxide occurred in the whole brain of exposed mice [61]. In a follow- up study, mice were again intranasally instilled every second day with the two types of TiO_2 _particles (80 nm, rutile or 155 nm, anatase; purity > 99%, about 500 μg per mouse, respectively). This time, brain tissues were collected at post-instillation time points of 2, 10, 20 and 30 days and evaluated for accumulation of TiO_2_, histopathology, oxidative stress, and inflammatory markers. It is shown in this study, that instilled TiO_2 _nanoparticles entered the brain directly through the olfactory bulb during the whole exposure period. In all brain parts and at all post-exposure time periods, the measured concentrations of both types of TiO_2 _particles were higher than in any of the controls. The anatase form of the TiO2 exhibited stronger effects on some of the investigated endpoints in both these studies. In the olfactory bulb, TiO_2 _contents increased gradually with time. TiO_2 _particles were mainly deposited in the hippocampus, where TiO_2 _contents were significantly increased after exposure for 2 days, then stayed constant for 10 and 20 days, before reaching the highest values after 30 days of exposure [64]. After 30 days of exposure, pathological changes were observed in olfactory bulb and hippocampus. Irregular arrangements of neurons in the olfactory nerve layers and dispersed arrangement and loss of neurons in the CA1 region of hippocampus were demonstrated. Hippocampal nerve cells were degenerated, together with changes in the nuclear membrane, mitochondria, rough endoplasmic reticulum, chromatin condensation, and elevated amounts of free ribosomes [64].

Shimizu et al [65] studied effects of anatase TiO_2 _(the particle size is not given in the article) that they injected subcutaneously (s.c.) into pregnant mice. Male embryos and pups were then investigated for certain gene expression patterns. The expression of genes associated with brain development, motor activity, oxidative stress, and apoptosis was changed compared to control animals during various periods of investigation (embryonic day 16 to 21 days post partum). Also Takeda et al [66] injected TiO_2 _(anatase, 25-70 nm) s.c. into pregnant mice. The nanoparticles were found in the brains (cortex, olfactory bulb) of the offspring. In addition, cells expressing the apoptosis marker Caspase-3 increased in the olfactory bulb of these animals. Abdominal injection of high dose anatase TiO_2 _(5 nm; 5-150 mg/g) to mice were performed daily for 14 days in a recent study [67]. The TiO_2 _content of the brains increased with increasing injection "doses". Also changes in neuronal morphology, transmitter levels and signs of oxidative stress were seen to follow a dose-response relationship.

The effects of various types of quantum dots (QDs) in the hippocampus of rats were investigated by Tang et al [68]. They found that both unmodified (CdSe) and modified (streptavidin-CdSe/ZnS) QDs can negatively affect synaptic transmission and plasticity in the rat hippocampus. The QDs were directly applied into the hippocampus and the effects on the electrophysiological properties of the neurons in the area were recorded after 20 min. Pair-pulse relation and long-term potentiation were significantly decreased after treatments. The effects were seen at two investigated concentrations of QD, 0.5 and 10 nM. The authors also reported that signs of oxidative stress were seen immediately after completion of the electrophysiological measurements. The results showed that SOD activity, GSH content and MDA levels all increased in the animals treated with QDs. These responses were stronger in the unmodified (CdSe) QDs, and more pronounced at the higher investigated concentration. This finding is possibly due to the toxic effects of Cd, which can be expected to be released from the unmodified QD. In a study by Maysinger et al [69] intracortical injection (μM concentrations) of various types of PEGylated QDs, non-PEGylated CdTe QDs, and CeO_2 _all caused activation of the glial cell marker GFAP to various degrees in mice. The effects were strongest in animals injected with CdTe QDs, and weakest after CeO_2 _treatment. Furthermore, the study showed that one of the PEGylated QDs, QD705, primarily accumulated in glia, whereas a small fraction (0.5%) could be found in neurons. Both these studies indicated that especially non-PEGylated QDs can cause inflammation and possibly gliosis in the brain. It is difficult to evaluate if the used concentrations are relevant for any real exposure situation.

In conclusion, the referenced studies point to that the investigated metallic nanoparticles all can translocate from the point of application (respiratory tract, skin, circulatory system) to the brains of the animals. However, it is unclear from these studies under which specific conditions this can be accomplished since the studies have not investigated dose-response relationships, properties of the ENPs in question etc. Certain of the observations are furthermore made in experiments where unrealistically high doses have been applied. A single study also indicates that a high dose of TiO_2 _can pass the placenta and be taken up into the brains of embryos. Regarding the physiological effects of these exposures, it is unclear to what extent, and at what exposure levels, nervous system functions can be affected by ENPs. However, the available data are suggestive of effects on neurotransmission, and possibly behaviour. Several signs of changes in oxygen radical homeostasis were also seen. The consequence of this could be that long-term exposures cause permanent inflammatory states, which can be a contributing factor in certain neurodegenerative diseases.

### In vitro studies

Synaptic transmission between neurons involves a number of structures and processes both in the pre- and the postsynaptic neuron. The presynaptic neuron needs to have the necessary machinery for synthesis of the neuron-specific transmitter. Furthermore, it is necessary to have structures for transmitter release and transmitter re-uptake or enzymatic degradation of transmitter. The post-synaptic neuron needs to express receptors for transmitters and together with its synaptic partner it has to express the structures that make the physical contacts in the synapse. To some extent, the development and function of neurotransmission in ENP exposed neurons have been investigated, along with studies on the toxicity of ENP on nervous system components.

Several studies have dealt with toxicity and oxidative stress due to ENP exposures. Thus, Pisanic et al. [70] showed that iron oxide ENP (5-12 nm) have cytotoxic effects on PC12 cells (a neuroendocrine cell line derived from rat pheochromocytoma). At 1.5 and 15 mM iron concentrations (but not at 0.15 mM) the ENP caused decreased cell viability. The response to NGF (nerve growth factor, inducing differentiation) in the PC12 cells was also negatively affected, seen as diminished neurite extension and number of neurites per cell. Also, the level of the GAP43 protein, a marker for neuronal differentiation, was decreased.

Maysinger et al. [69] showed that certain QDs were taken up into differentiated PC12 cells, whereas other (non-PEGylated) QDs caused cell death. Increased oxidative stress, measured as H_2_O_2 _production, was seen in immortalized microglia cells exposed to Degussa P25 ENP that formed aggregates. The doses (2.5-120 ppm) of the P25 aggregates were non-cytotoxic [71]. Alekseenko and coworkers [72] used rat brain synaptosomes to test ferritin molecules that contain Fe^3+ ^iron particles (7 nm). The treatment caused ROS formation at high doses (800 μg/ml), whereas the effects of 80 and 8 μg/ml were not significantly different from controls. The higher concentration could not induce glutamate release, but inhibited uptake of glutamate. Consequently, at 800 μg/ml, iron-based nanoparticles can cause conditions that can lead to neurodegeneration. Schubert et al. [73] showed that both CeO ENPs (6 and 12 nm) and YO ENPs (12 nm) are neuroprotective in cultured hippocampal neurons (HT22 cell line). The cells were treated with glutamate to generate ROS at levels that were cytotoxic, which was counteracted by addition of the mentioned ENPs.

Conflicting results are available regarding the effects on cytotoxicity and oxidative stress from fullerenes (C_60_). Sayes et al. [74] used a water-soluble fullerene species, nano-C_60 _that was cytotoxic to several human cell types, including astrocytes. The cytotoxicity was mediated by lipid peroxidation according to the authors. On the other hand, polyhydroxylated C_60 _fullerenols at μM concentrations acted as antagonists to glutamate receptors in a study by Jin et al. [75]. The C_60 _particles blocked primarily the AMPA-type glutamate receptor in neuronal cultures from rat brain, and also to some extent NMDA and KA receptors. The antagonistic behaviour on glutamate receptors were not seen in GABA or taurine receptors. In the absence of C_60_, higher concentrations of glutamate were needed to elicit similar effect. The fullerenes could also act as antioxidants, inhibiting effects of added H_2_O_2 _and Fe^2+^. An earlier study by Dugan et al. [76] also showed neuroprotective effects of C_60 _fullerenes on cortical cell cultures exposed to NMDA or AMPA at concentrations that caused excitotoxicity.

The effects of Mn ENP on transmitter levels in PC12 cells were seen by Hussain et al. [77]. The Mn nanoparticles specifically caused depletion of dopamine stores in the PC12 cells. This occurred in a dose-dependent fashion (concentrations ranging from 1-100 μg/ml) after cells were exposed for 24 h to 40 nm particles. The effect was similar to effects of added Mn^2+ ^ions. However, the levels of ROS were much higher after Mn ENP addition compared to Mn^2+^, or compared to Ag ENP (15 nm). The latter ENP also caused dopamine depletion, although to a lesser extent than Mn ENP.

In two studies Wang et al. have documented that ENP inhibit the acetylcholine degrading enzymes acetylcholine esterase [78] and butyrylcholine esterase [79] in solution. Several different types of ENP (MWCNT, SWCNT, Cu, TiO_2_) could adsorb and thus inhibit enzyme activities in a dose-dependent fashion. The authors suggested that the inhibitory effects were caused by ion dissolution from the ENPs.

The communication between neurons relies on transmitter release which in turn is dependent on changes in ion concentrations on the in and outside of the neuronal membrane. Since such changes lead to displacement of charged entities, it is possible to measure these events by analyzing electric potentials that are present over the membrane. Several studies have investigated whether currents that pass through ion specific membrane channels are affected by ENP.

Tang et al. [68] studied the effects of CdSe QDs (2.38 nm) on primary cultures of rat hippocampal neurons. At 10 nM or higher concentrations, these particles caused cell death, due to sustained increases in intracellular Ca^2+ ^levels. The particles also had effects on voltage gated Na-channels, where patch-clamp analyses revealed enhanced activation and inactivation of the sodium current, and also a prolonged activation time and increased recovery time for the Na^2+ ^current. Thus, fewer Na-dependent potentials would occur in these cells, interfering with normal synaptic transmission. Another study revealed that silver particles (244.4 nm; 12.5 m^2^/g) could inhibit Na^+ ^currents in rat hippocampal slices [80]. This occurred at 10 μg/ml, but not at lower concentrations. Zhao et al [81] could show that ZnO ENP (20-80 nm; 2-3 crystal forms; 100 μg/ml, but not at lower concentrations), increased amplitudes of both Na^+ ^and K^+ ^currents, by increasing the number of open Na^+ ^channels, delaying rectifier K^+ ^channels and thus enhancing the excitability of neurons. Xu et al. [82] have shown that CuO ENP (60.6 nm; 15.7 m^2^/g; 50 μg/ml) could inhibit the rectifier K^+ ^current in rat CA1 pyramidal hippocampus neurons. Jakubek et al. [83] demonstrated that carbon nanotubes could inhibit the function of Ca^2+ ^channels expressed in human embryonic kidney tsA201 cells. This effect was probably due to the release of Ni^+ ^and Y^+ ^ions from the carbon nanotubes, and that these ions displaced Ca^2+ ^from the channel pore.

Taken together, these findings give some support for the concept that several types of ENPs under specific in vitro conditions can influence the electrophysiological properties of neurons.

Exposure to silver is likely to increase due to the increased use of silver nanoparticles. A recent study [84] asked the question if silver ions (AgNO_3_) can have effects on the developing nervous system. The reason why the authors investigated silver ions was that silver nanoparticles are releasing ions according to the authors. The experimental model was the mouse PC12 neuroblastoma cell line which can be induced to differentiate into neurons in the presence of NGF (nerve growth factor). The cells were exposed for 1 h to Ag^+ ^at 1 or 10 μM, or to control substances (chlorpyrifos, which is a known developmental neurotoxicant; NaNO_3 _to investigate if effects were due to NO_3_^- ^ions or to Ag^+^). In undifferentiated cells, both concentrations of Ag^+ ^inhibited DNA and protein synthesis. The higher concentration furthermore caused cell death and oxidative stress, to an extent which was larger than the positive control chlorpyrifos. Furthermore, it was clear from the experiments that it was the Ag^+ ^ion and not the NO_3_^- ^that was responsible for the effects. Continuous exposure to Ag^+ ^in cells that were induced to differentiate caused DNA synthesis inhibition and oxidative stress, and also inhibition of the differentiated phenotype (dopaminergic neurons), whereas cholinergic neuron differentiation was favoured. This study suggests that Ag^+ ^can exert a developmental neurotoxic effect at higher concentrations that are even stronger than a known neurotoxicant. Also at the lower Ag^+ ^level, effects were present, although less pronounced. However, one has to keep in mind that this study was not dealing with silver nanoparticles but instead was designed on the assumption that silver ENPs would act as a depot for release of silver ions. In the mouse neural stem cell line C17.2, TiO_2 _ENP (50-250 μg/ml; 80-100 nm; rutile form) could lower the proliferation rate and induce neuronal differentiation [85]. The authors also performed protein expression profiling and found that the induction of differentiation by TiO_2 _was accompanied by changes in the levels of nine of the investigated proteins. These data suggest that TiO_2 _effects include modulation of the PKC-epsilon pathway.

A common feature for several neurodegenerative diseases is the formation of extra- or intracellular protein complexes or aggregates. In e.g. Alzheimer's disease, the so-called amyloid hypothesis states that aggregates of the beta-amyloid peptide are neurotoxic and cause local inflammations that are detrimental for neurons. The reasons for formation of these aggregates are manifold, including both genetic and environmental factors. Since so many patients are diagnosed with diseases like Alzheimer's every year, there is a constant interest into potential aggravating factors. Thus, the question is if ENPs can trigger or promote formation of beta-amyloid aggregates. Wu et al. [86] have seen that TiO_2 _ENP (20 nm; 80:20 anatase: rutile) in a concentration dependent manner (4-20 μM) accelerates the fibrillation, and thus aggregate formation, of the beta-amyloid peptide in solution. The proposed mechanism is that the nucleation process, which is rate limiting for fibril formation, is shortened. Also other ENP were previously seen to stimulate protein fibril formation. Thus, several types of nanoparticles (copolymer particles, cerium oxide particles, QDs, carbon nanotubes) stimulated faster formation of fibrils of the beta_2_-microglobulin protein [87]. Both these studies thus suggest that formation of potentially neurotoxic protein fibrils can be enhanced by ENPs. However, these studies have to be treated with caution, since the experiments were performed in solution, and not in any living system. Whether this is relevant for any in vivo situation is unclear (Table 2).

## Risk assessment and research needs

**Figure 3:**
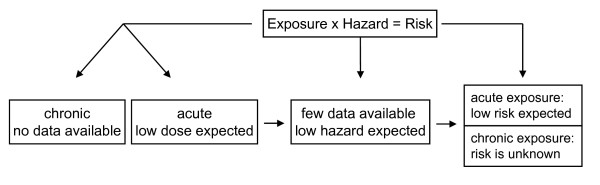
**Risk assessment of ENPs to the brain has to be considered for both acute and chronic exposure**. The risk due to acute exposure of ENPs is expected to be low based on current knowledge. The lack of appropriate studies for chronic exposure makes it impossible to assess the risk at present. A detailed discussion is given in the text.

A health risk assessment has to consider data from various lines of evidence (e.g. human epidemiological and clinical studies, experimental animal and in vitro studies, in silico studies) and integrate these into a cohesive evaluation. It is furthermore essential to have relevant information on exposure. A risk can then be deduced from exposure data together with the hazard assessment. Needless to say, the assessment becomes more reliable when more relevant information is available. Here we try to make an assessment of the risks for neurological effects in humans that are subjected to unintended air-borne exposures (i.e. non-clinical) of ENP (see also Figure 3).

Data on assessment of human exposure to ENPs is very sparse. However, there is at present very little reason to expect that the general public is exposed to any significant amounts of air-borne ENPs, although ENPs are present in certain consumer products. It is more likely that occupational exposures can be a factor in at least some settings.

Besides the few data on exposure that makes risk assessment difficult, the absence of a relevant dose concept for quantification of hazards is an obstacle. We consider this deficiency to be one of the biggest problems for risk assessment of ENPs today. There are different models available to study toxicological effects of nanomaterials in the human body, like physiologically-based pharmacotoxic and pharmacokinetic models, but in addition the experience from radiobiology in generating dose concepts could be very valuable. Thus, knowledge about the retention time of the nanomaterial in the body, and also half-life, (cf. radionuclides that have dual effects; the effects of the element itself plus the effect of the irradiation that the nuclide generates) is essential to get an idea of both dose and unintended reactions in vivo. Furthermore, the dose rate (the kinetics of the uptake of ENPs per unit of time (acute high dose exposure vs. chronic low dose exposure)) is an important aspect of exposure, as well as the ENPs physicochemical structure.

Even during clinical situations, where ENPs are created to act as drug delivery systems, translocation to CNS is difficult to assess. It has been shown that special coverings and functional modifications of the surface of ENPs are necessary for them to reach the target organ, in this case the CNS.

Also experimental studies on animals suggest that translocation even after instillation or inhalation of substantial amounts is very low, but can occur (see also Figure 2 for an overview of translocation routes). The knowledge regarding the specific physico-chemical characteristics that are important for translocation is sparse. It is feasible that also in humans, translocation to at least some degree can occur as a consequence of environmental and/or occupational exposure. Importantly, there are no long term data available which could demonstrate chronic exposure conditions. It has to be pointed out that chronic exposure is relevant for non-biodegradable and non-excreted ENPs, which can accumulate over time within the brain leading to long term (toxic) effects. In addition, long term and low "dose" exposure to biodegradable ENPs can induce chronic inflammation-like conditions by oxidative stress. Such a condition can lead to pathological processes in the CNS. Chronic exposure to ENPs within the CNS could possibly also aggravate ongoing pathological processes. Regrettably, this is presently only speculation since knowledge about the effects of chronic and long term/low dose exposure is entirely missing.

If ENPs are reaching the CNS through the olfactory nerve after inhalation, the numbers of particles (dose) can be higher (acute exposure) then by translocation through the lungs. This circumstance can be relevant for occupational exposure. Also chronic exposure in occupational settings can lead to a brain exposure, both through the lung and/or the olfactory nerve. However, if a high CNS-exposure would occur, other parts of the body would experience even higher exposures and thus stronger toxic effects.

Table 2 summarizes in vivo and vitro data on effects caused by ENPs on properties and functions of the CNS. The noted effects suggest that several types of ENPs can have various types of biological effects. Some of these effects could also imply that ENPs can cause hazards, both in an acute fashion and in the long term. However, the relevance of these data for risk assessment is far from clear. At issue is especially if these effects would occur at levels that are relevant for environmental or occupational exposure.

Since investigations into the possible harmful effects of ENP have been performed only for a few years, it is not surprising that many studies suffer from shortcomings. It is nevertheless the view of these authors that it is possible to improve the quality of the studies with a few means, as outlined below.

It is essential

• that exposure assessments are performed so that experimental studies can investigate effects of specific ENPs at relevant "doses". Some of the presently available studies are using enormously high doses of ENPs without any relevance for risk assessment. However, it should also be mentioned that also exposures to high doses can be informative, especially in the identification of possible hazards

• to perform more dose-response studies

• to use appropriate controls and studies should be performed in a blinded manner. Very often the informative value of a study would vastly improve if, for toxicology studies, a relevant positive control was applied. Positive controls are furthermore essential for validation of the methodology used. Admittedly, positive controls are sometimes difficult to identify, but are basically physical or chemical agents with known mechanisms of action.

• to correctly describe the physico-chemical properties of the ENP, which is sometimes missing. Essential information includes data on size, shape and composition (which includes surface charge and adsorbed species) and also redox-reactivity.

• to have knowledge about possible surface modifications, whether the ENP aggregates and their dissolution or degradation is needed (see e.g. [82] for details).

• for risk assessment to report negative findings.

For adequate risk assessment of chronic exposure, information about metabolism of ENPs within the CNS, accumulation, dose definition etc is needed. Obviously, at the present state of knowledge, the risk assessment needs to be performed on a case by case basis.

## Conclusion

The aim of the present study is to assess if there is a risk to especially the CNS after unintended exposure to inhaled ENPs. A possible risk has two components, viz. exposure and hazard. Regarding exposure, there are at present very few if any data on exposure of the general public to either acute high dose exposure or on chronic exposure to low dose levels of air-borne ENPs. It is furthermore unlikely, with the exception of possibly a few occupational situations that acute high dose exposures would happen. The risks from such exposures for damaging CNS effects is thus probably very low, irrespective of any biological hazards that ENPs could constitute.

The situation is more complicated regarding chronic exposures, at low doses. The long term accumulation of ENPs can not be excluded. However, we do not have access to exposure data for the general public regarding ENPs. We also know that translocation to the brain via respiratory organs and the circulation is very low, even in cases where ENPs have such surface modifications as to be able pass the BBB. At higher concentrations, ENP can possibly enter the olfactory bulb via the olfactory nerve, and then possibly distribute to other areas of the brain. It is also shown in both in vivo and in vitro studies that several types of ENP have various types of biological effects. The relevance of these data is unclear. However, a possibility remains that chronic exposures, and/or biopersistent ENPs, can influence processes within the brain that are triggering or aggravating pathological processes.

In general, the present state of knowledge is unsatisfactory for a proper risk assessment in this area. Improvements of the study qualities as well as increased number of relevant studies are strongly recommended.

## Competing interests

The authors declare that they have no competing interests.

## Authors' contributions

MS conceived of the study and participated in data collection and screening, data analysis, drawing of conclusions. MOM participated in data collection and screening, data analysis, drawing of conclusions. Both authors drafted the manuscript, read and approved the final manuscript.
